# Supplementary material for: Environmental microbial community signatures associated with Listeria spp. detection in German meat processing facilities
Source: Appl Environ Microbiol. 2026 Aug 28;92(9):e01042-26. doi: 10.1128/aem.01042-26 (PMC13599490; doi:10.1128/aem.01042-26)
Supplement: Supplementary file 1 [file aem.01042-26-s0006.pdf]

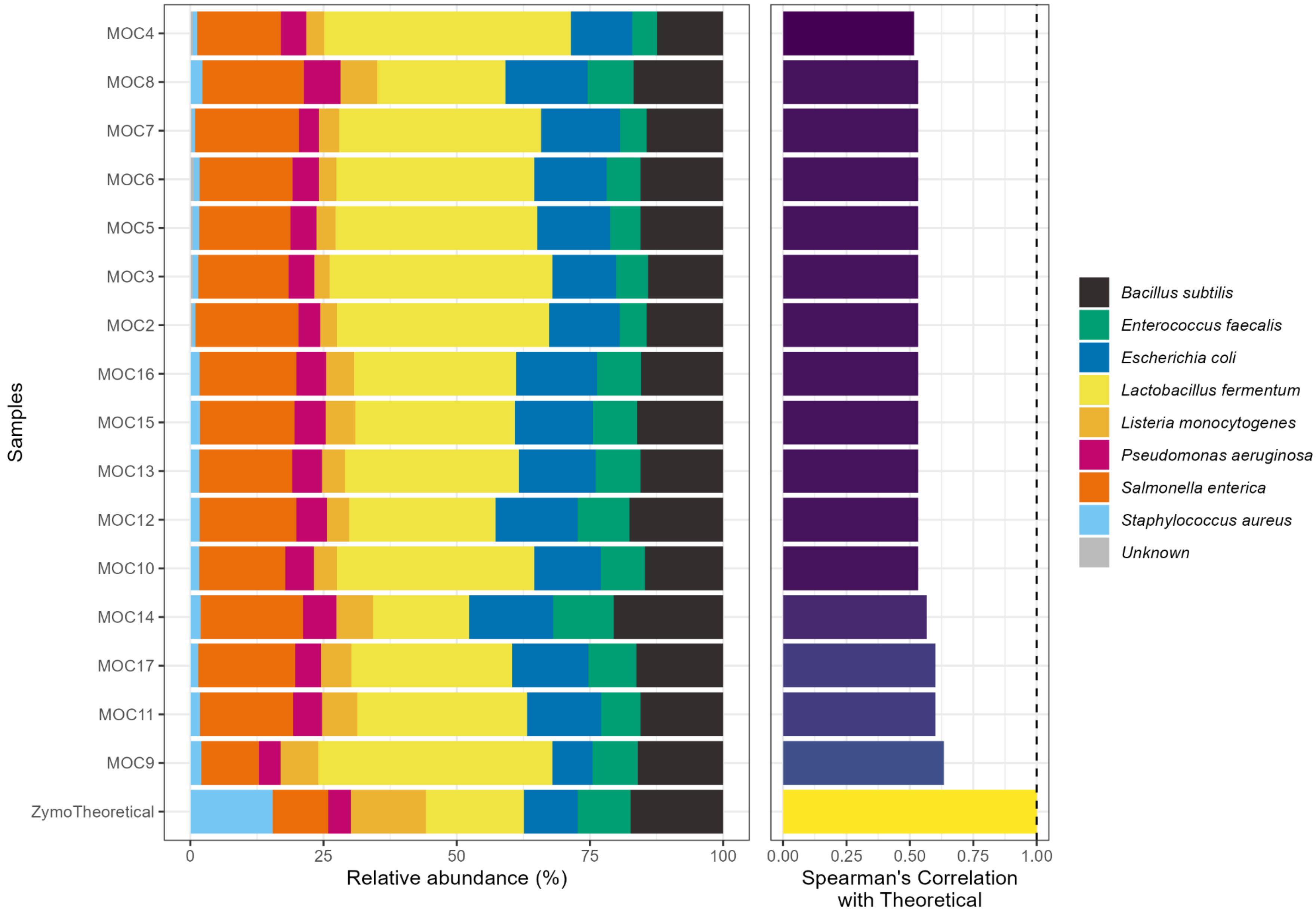

Fig. S1. Mock community composition across extraction runs. Stacked bar plots show the observed relative abundances of the expected taxa in mock community samples across extraction runs, together with the theoretical composition of the standard. The right panel shows Spearman's correlation coefficients between the observed and theoretical profiles. All expected taxa were recovered in each run, and deviation patterns were consistent across runs.

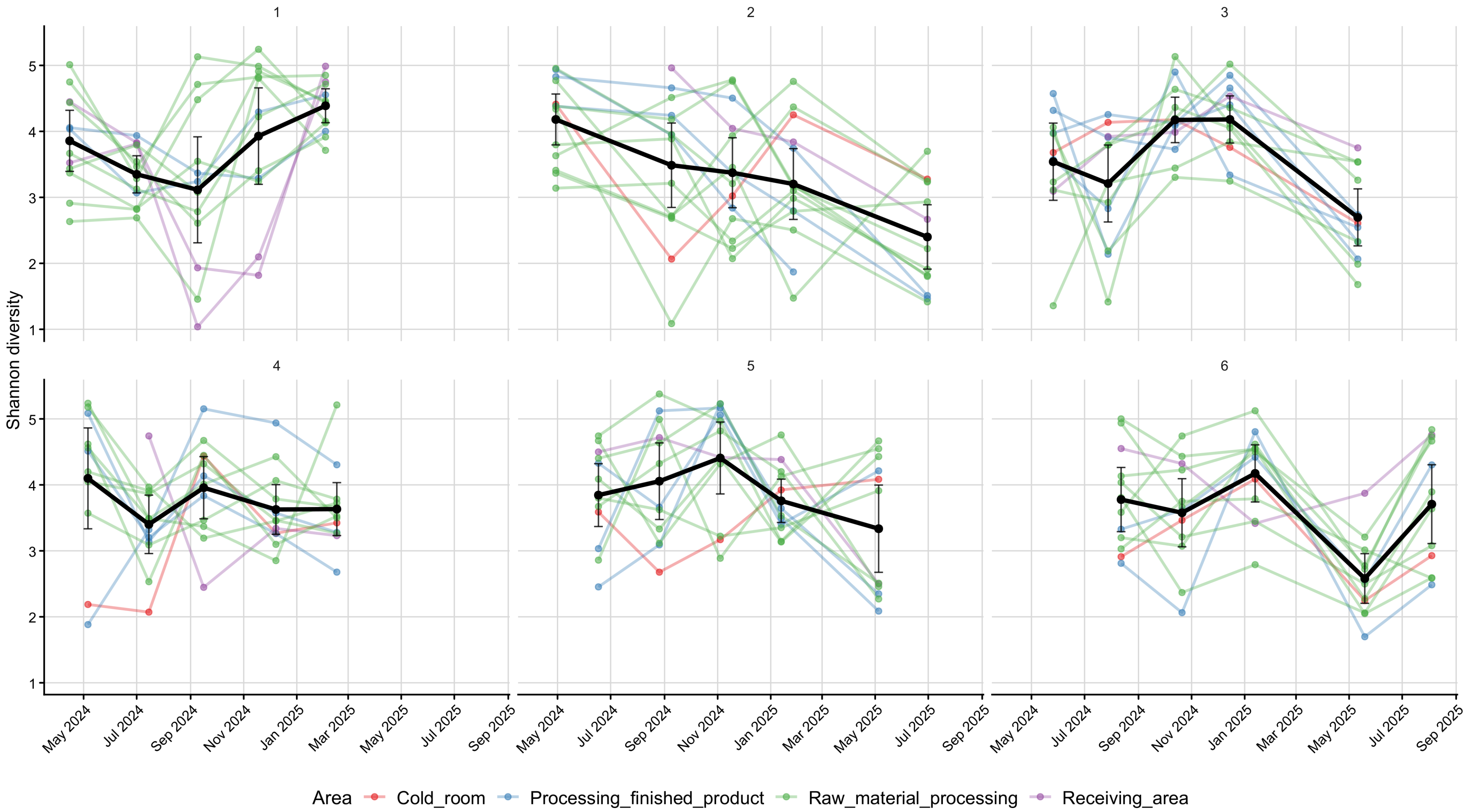

Fig. S2. Facility-stratified temporal patterns of Shannon diversity. Shannon diversity is shown for samples from each facility across the study period. Points represent individual observed samples, and thin colored lines connect repeated observations from the same sampling site. Colors denote production areas. Black points and lines show observed facility-level means per sampling event; error bars represent 95% confidence intervals. Statistical testing was performed using mixed-effects models with sampling event modeled as a categorical predictor to assess temporal variation without assuming a linear trend.

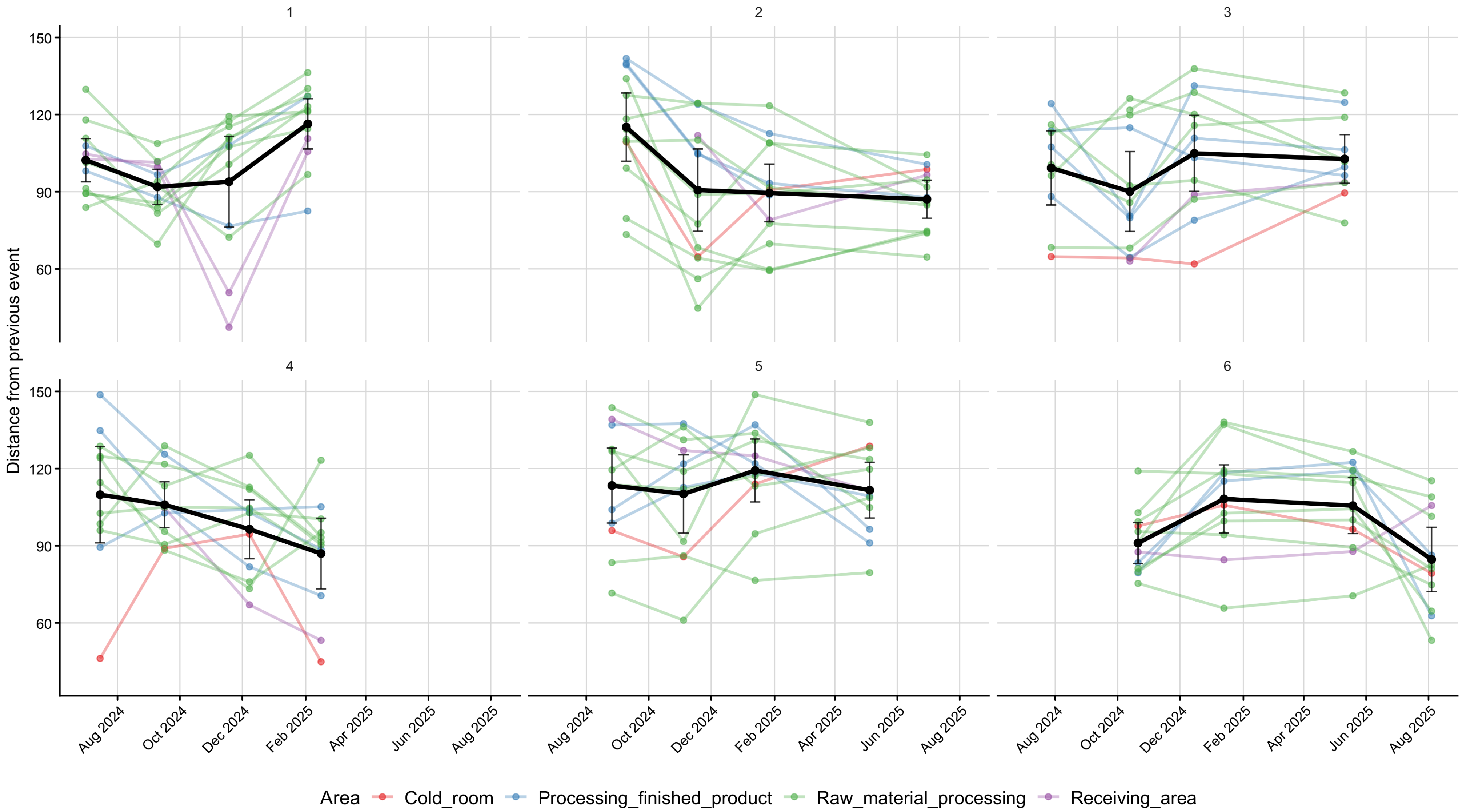

Fig. S3. Facility-stratified patterns of longitudinal changes in community composition. Longitudinal community change was quantified as the Aitchison distance between samples from the same sampling unit at consecutive sampling events. Points represent individual observed sampling units, and thin colored lines connect repeated observations from the same sampling unit. Colors denote production areas. Black points and lines show observed facility-level means per sampling event; error bars represent 95% confidence intervals. Statistical testing was performed using mixed-effects models with sampling event modeled as a categorical predictor to assess temporal variation without assuming a linear trend.
