# Supplementary material for: Environmental microbial community signatures associated with Listeria spp. detection in German meat processing facilities
Source: Appl Environ Microbiol. 2026 Aug 28;92(9):e01042-26. doi: 10.1128/aem.01042-26 (PMC13599490; doi:10.1128/aem.01042-26)
Supplement: Supplementary file 2 [file aem.01042-26-s0007.docx]

Supplementary Microbiome Data Analyses

# Table S1. Core genera by facility

Core genera were defined as genera present in at least 90% of samples from a given facility at a minimum relative abundance of 0.0001. Mean and standard deviation refer to relative abundance values.

| **Facility** | **Genus** | **Mean relative abundance** | **SD** |
| --- | --- | --- | --- |
| F1 | Acinetobacter | 0.157591 | 0.19730314 |
| F1 | Chryseobacterium | 0.03787727 | 0.05948863 |
| F1 | Janthinobacterium | 0.0325039 | 0.08149922 |
| F1 | Kocuria | 0.11866455 | 0.17632307 |
| F1 | Pseudomonas | 0.1830002 | 0.24685973 |
| F1 | Rhodococcus | 0.04645399 | 0.0879744 |
| F2 | Acinetobacter | 0.2135272 | 0.2930908 |
| F2 | Pseudomonas | 0.1610189 | 0.2084982 |
| F3 | Acinetobacter | 0.21502456 | 0.2645593 |
| F3 | Microbacterium | 0.05860628 | 0.1329138 |
| F3 | Pseudomonas | 0.17162529 | 0.2118463 |
| F4 | Acinetobacter | 0.09700433 | 0.18803645 |
| F4 | Aeromonas | 0.02371492 | 0.05072769 |
| F4 | Pseudomonas | 0.23589677 | 0.29177973 |
| F5 | Acinetobacter | 0.219813002 | 0.2696412 |
| F5 | Aeromonas | 0.018199969 | 0.04112017 |
| F5 | Chryseobacterium | 0.008919896 | 0.02235674 |
| F5 | Flavobacterium | 0.011873828 | 0.03009697 |
| F5 | Janthinobacterium | 0.006330362 | 0.01230215 |
| F5 | Pseudomonas | 0.153751574 | 0.15917931 |
| F6 | Acinetobacter | 0.132126121 | 0.14252422 |
| F6 | Enhydrobacter | 0.018476131 | 0.03237862 |
| F6 | Exiguobacterium | 0.01922357 | 0.03310487 |
| F6 | Kocuria | 0.08495531 | 0.1417309 |
| F6 | Microbacterium | 0.036248691 | 0.08090281 |
| F6 | Paracoccus | 0.009147466 | 0.01973553 |
| F6 | Pseudomonas | 0.065772798 | 0.09291492 |
| F6 | Psychrobacter | 0.046898869 | 0.08423064 |
| F6 | Rhodococcus | 0.061790572 | 0.13243219 |
| F6 | Rothia | 0.087986749 | 0.13563307 |

# Table S2. Core genera across all facilities

Overall core genera were identified using the same threshold across all samples.

| **Genus** | **Mean relative abundance** | **SD** |
| --- | --- | --- |
| Acinetobacter | 0.173501 | 0.2367193 |
| Pseudomonas | 0.1615972 | 0.2156033 |

# Table S3. Core ASVs by facility

Core ASVs were defined using the same prevalence and relative-abundance criteria as above. No core ASVs were identified in F2 or F3.

| **Facility** | **ASV** | **Genus** | **Mean relative abundance** | **SD** |
| --- | --- | --- | --- | --- |
| F1 | ASV1 | Rhodococcus | 0.02321436 | 0.0288483 |
| F1 | ASV7 | Kocuria | 0.06051748 | 0.10109978 |
| F1 | ASV26 | Janthinobacterium | 0.0129656 | 0.03721952 |
| F1 | ASV64 | Pseudomonas | 0.00959209 | 0.01982775 |
| F2 | None identified | - | - | - |
| F3 | None identified | - | - | - |
| F4 | ASV42 | Pseudomonas | 0.00942408 | 0.02188447 |
| F5 | ASV16 | Pseudomonas | 0.01006089 | 0.02036958 |
| F6 | ASV1 | Rhodococcus | 0.05941303 | 0.13175498 |
| F6 | ASV11 | Rothia | 0.06295537 | 0.09594127 |
| F6 | ASV14 | Acinetobacter | 0.00950572 | 0.01716584 |
| F6 | ASV25 | Enhydrobacter | 0.01720186 | 0.0272359 |

# Table S4. Pairwise PERMANOVA comparisons among facilities

Pairwise PERMANOVA was used to compare microbial community composition between facilities. The table reports the facility term for each pairwise comparison.

| **Comparison** | **Df** | **SumOfSqs** | **R2** | **F** | **p value** |
| --- | --- | --- | --- | --- | --- |
| F1 vs F5 | 1 | 31330 | 0.05256 | 6.6428 | 0.001 |
| F1 vs F4 | 1 | 30153 | 0.05163 | 6.4659 | 0.001 |
| F1 vs F3 | 1 | 30177 | 0.05351 | 6.7239 | 0.001 |
| F1 vs F6 | 1 | 45256 | 0.07938 | 10.3489 | 0.001 |
| F1 vs F2 | 1 | 29170 | 0.05082 | 6.8607 | 0.001 |
| F5 vs F4 | 1 | 27842 | 0.04725 | 5.8024 | 0.001 |
| F5 vs F3 | 1 | 18540 | 0.03312 | 4.0079 | 0.001 |
| F5 vs F6 | 1 | 35225 | 0.06206 | 7.8074 | 0.001 |
| F5 vs F2 | 1 | 20374 | 0.03558 | 4.6478 | 0.001 |
| F4 vs F3 | 1 | 26015 | 0.04675 | 5.6892 | 0.001 |
| F4 vs F6 | 1 | 43149 | 0.07641 | 9.6792 | 0.001 |
| F4 vs F2 | 1 | 30815 | 0.05384 | 7.1131 | 0.001 |
| F3 vs F6 | 1 | 25502 | 0.0484 | 5.9507 | 0.001 |
| F3 vs F2 | 1 | 12114 | 0.02271 | 2.9045 | 0.001 |
| F6 vs F2 | 1 | 33725 | 0.06174 | 8.2915 | 0.001 |

# Table S5. PERMANOVA model for L. monocytogenes presence

Model: Aitchison distance ~ L. monocytogenes * Facility * Area; 999 permutations.

| **Term** | **Df** | **SumOfSqs** | **R2** | **F** | **p value** |
| --- | --- | --- | --- | --- | --- |
| Listeria monocytogenes | 1 | 14625 | 0.00834 | 3.4665 | 0.001 |
| Facility | 5 | 143286 | 0.0817 | 6.7923 | 0.001 |
| Area | 3 | 57054 | 0.03253 | 4.5076 | 0.001 |
| Listeria monocytogenes:Facility | 4 | 16502 | 0.00941 | 0.9778 | 0.548 |
| Listeria monocytogenes:Area | 3 | 14058 | 0.00802 | 1.1107 | 0.224 |
| Facility:Area | 14 | 95754 | 0.0546 | 1.6211 | 0.001 |
| Listeria monocytogenes:Facility:Area | 4 | 15906 | 0.00907 | 0.9425 | 0.659 |
| Residual | 331 | 1396515 | 0.79632 |  |  |

# Table S6. PERMANOVA model for Listeria spp. presence

Model: Aitchison distance ~ Listeria spp. * Facility * Area; 999 permutations.

| **Term** | **Df** | **SumOfSqs** | **R2** | **F** | **p value** |
| --- | --- | --- | --- | --- | --- |
| Listeria spp. | 1 | 24054 | 0.01372 | 5.6806 | 0.001 |
| Facility | 5 | 141833 | 0.08088 | 6.6992 | 0.001 |
| Area | 3 | 52655 | 0.03002 | 4.1451 | 0.001 |
| Listeria spp.:Facility | 5 | 24295 | 0.01385 | 1.1475 | 0.070 |
| Listeria spp.:Area | 3 | 14671 | 0.00837 | 1.1549 | 0.096 |
| Facility:Area | 14 | 91309 | 0.05207 | 1.5403 | 0.001 |
| Listeria spp.:Facility:Area | 9 | 28728 | 0.01638 | 0.7538 | 1.000 |
| Residual | 325 | 1376156 | 0.78472 |  |  |

# Table S7. Logistic regression of culture-based Listeria spp. detection against Listeria ASV abundance

Culture positivity was modeled as the binary response variable and sequencing-based Listeria ASV abundance as the predictor.

| **Term** | **Estimate** | **Std. error** | **z value** | **p value** | **OR** | **OR 2.5%** | **OR 97.5%** |
| --- | --- | --- | --- | --- | --- | --- | --- |
| Intercept | -1.7084127 | 0.1586982 | -10.765 | <2e-16 | 0.1811531 | 0.1313704 | 0.2450540 |
| Listeria ASV abundance | -0.0012057 | 0.0008621 | -1.399 | 0.162 | 0.9987950 | 0.9964636 | 0.9999289 |

# Table S8. ASVs positively associated with Listeria spp.-positive drain samples

Differential abundance analysis was conducted on drain samples. Associations were considered significant at an FDR-adjusted threshold of q < 0.1. No ASVs were significant in the prevalence model and no ASVs were significantly associated with L. monocytogenes status after FDR correction.

| **Genus** | **ASV** | **Model** | **Beta coefficient** | **q value** |
| --- | --- | --- | --- | --- |
| Acinetobacter | ASV167 | Abundance | 6.50 | 0.064 |
| Rhizorhapis | ASV2147 | Abundance | 4.19 | 0.029 |
| Vagococcus | ASV447 | Abundance | 2.82 | 0.029 |
